# Erythrasma Revisited: Diagnosis, Differential Diagnoses, and Comprehensive Review of Treatment

**DOI:** 10.7759/cureus.10733

**Published:** 2020-09-30

**Authors:** Parnia Forouzan, Philip R Cohen

**Affiliations:** 1 Dermatology, McGovern Medical School, University of Texas Health Science Center at Houston, Houston, USA; 2 Dermatology, San Diego Family Dermatology, National City, USA

**Keywords:** corynebacterium, diagnosis, differential, erythrasma, infection, lamp, minutissimum, mupirocin, treatment, wood

## Abstract

Erythrasma is a bacterial infection of the skin typically caused by Corynebacterium minutissimum. This pathogen infects the stratum corneum in warm and wet areas of the skin. Most commonly, the axillary, inguinal, and interdigital regions are affected. A 60-year-old man presented for the examination of a pedunculated lesion on his right proximal thigh. Upon examination of the lesion, adjacent areas of central hypopigmentation and peripheral hyperpigmented scaling were also noted bilaterally in the groin region. Differential diagnoses of candidiasis, dermatophyte infection, erythrasma, pityriasis versicolor, and terra firma-forme dermatosis were considered. Wood lamp examination revealed bright coral-pink fluorescence. Correlation of the clinical examination and the Wood lamp finding established the diagnosis of erythrasma. Twice daily topical 2% mupirocin ointment therapy led to the resolution of our patient’s erythrasma. In this case report, the diagnosis, differential diagnoses, and treatment of erythrasma are reviewed.

## Introduction

Erythrasma is a bacterial infection of the skin caused by *Corynebacterium minutissimum*. *C. minutissimum* is a gram-positive bacterium most commonly found in warm, moist regions of the skin. Specifically, this pathogen is found in the stratum corneum, the uppermost layers of the epidermis [1]. Erythrasma primarily occurs in skin folds such as in the axillary and inguinal regions. It can also be found on toe webs (interdigital erythrasma) and as a coinfection with *Candida albicans* or dermatophytes (*Epidermophyton, Microsporum, *and* Trichophyton*). The clinical presentation of erythrasma can resemble candidiasis, dermatophyte infections, pityriasis versicolor, and terra firma-forme dermatosis [1, 2].

A 60-year-old man presented for removal of a pedunculated lesion from his right proximal medial thigh. Clinical examination also revealed adjacent asymptomatic plaques with central hypopigmentation and peripheral hyperpigmented scaling bilaterally in the groin region. Bright coral-pink fluorescence was demonstrated on Wood lamp examination. The Wood lamp finding aided in establishing the diagnosis of erythrasma. The patient’s skin infection was successfully treated with twice daily topical application of 2% mupirocin ointment for six weeks. The diagnosis, differential diagnoses, and management of erythrasma are reviewed.

## Case presentation

**Figure 1:**
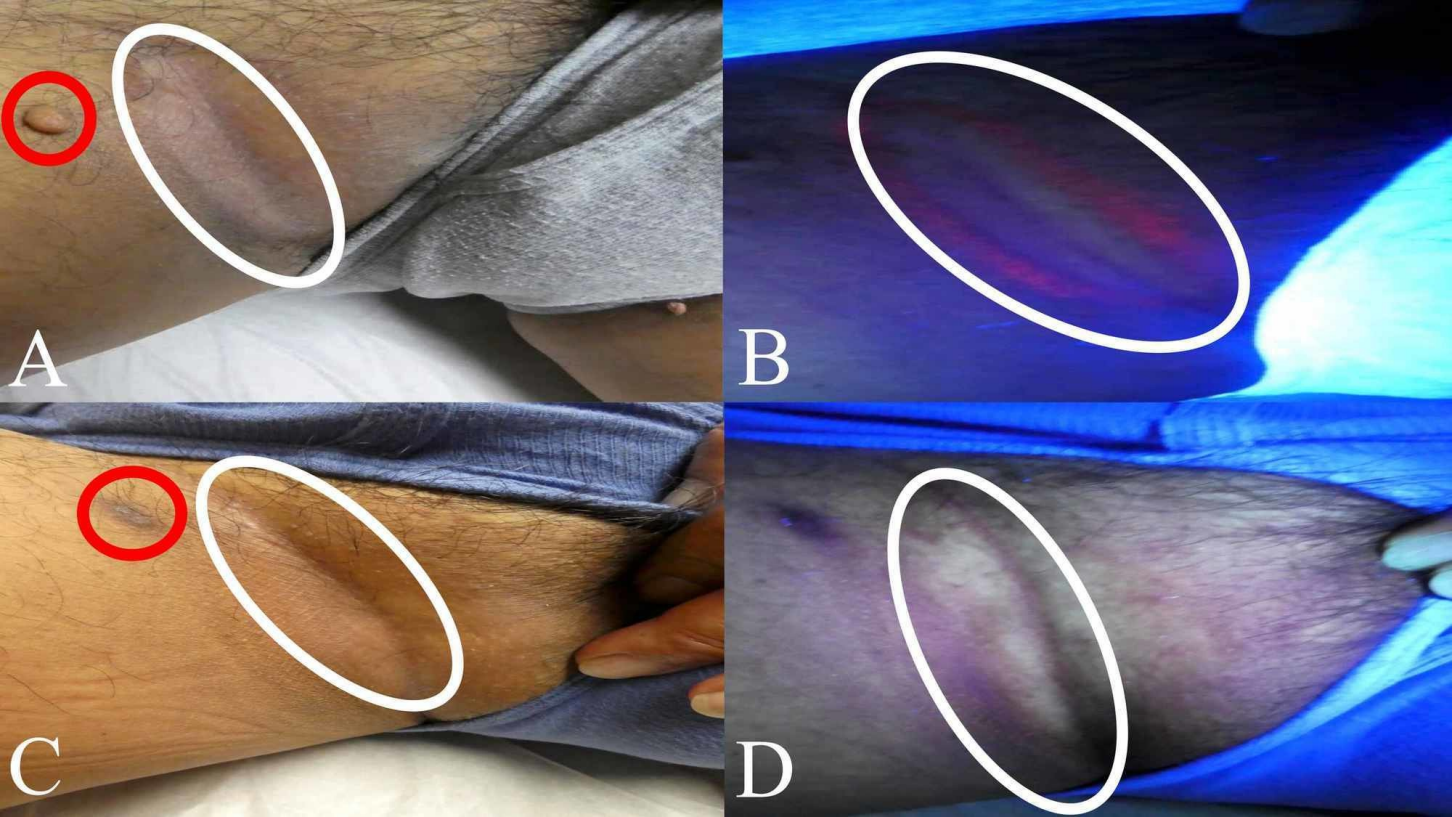
Clinical presentation of erythrasma on the right inguinal fold A 60-year-old man presented for a skin check. He was concerned about a pedunculated lesion on his right proximal thigh (circled in red). Clinical examination also showed a central area of hypopigmentation with peripheral hyperpigmentation and scaling (circled in white) on his right inguinal fold (A). Wood lamp examination revealed a bright coral-pink fluorescence characteristic of erythrasma on the right inguinal fold (B). After six weeks of twice daily topical application of 2% mupirocin ointment, the biopsy site had healed (circled in red), and his previous lesion of erythrasma (circled in white) had resolved (C). Wood lamp examination was negative for erythrasma on his right inguinal fold; there was no residual coral-pink fluorescence (circled in white) after six weeks of topical 2% mupirocin ointment therapy (D).

A 60-year-old Hispanic man with Fitzpatrick skin type IV presented for a skin examination; he had light brown skin that minimally burned, and he tanned easily. The patient was concerned about a flesh-colored, pedunculated lesion on his right proximal medial thigh (Figure 1). A skin biopsy was performed, and pathology evaluation diagnosed a benign nevus lipomatosus.

**Figure 2:**
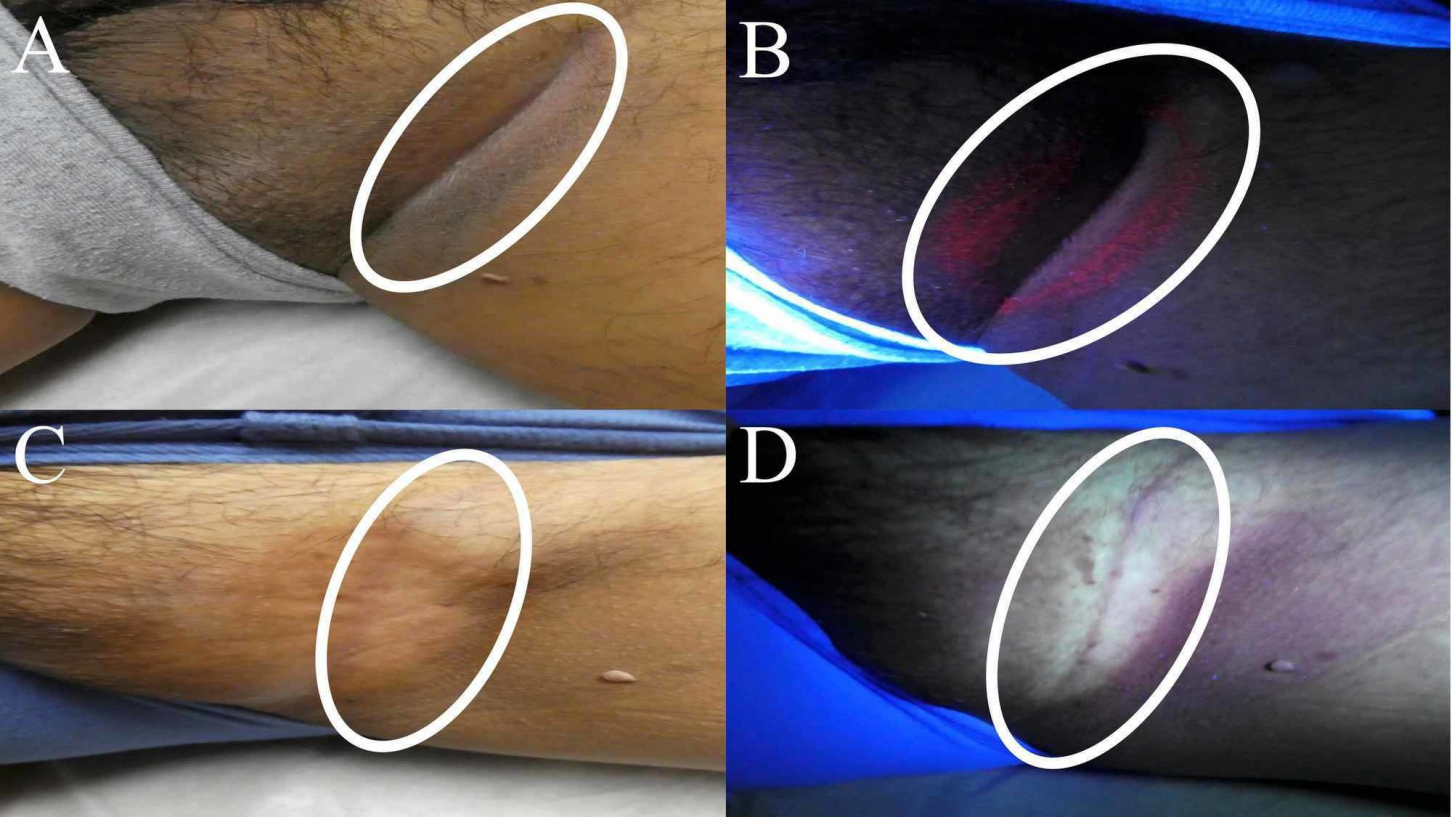
Clinical presentation of erythrasma on the left inguinal fold Clinical examination also showed a central area of hypopigmentation with peripheral hyperpigmentation and scaling (circled in white) on his left inguinal fold (A). Wood lamp examination revealed a bright coral-pink fluorescence characteristic of erythrasma on the left inguinal fold (B). After six weeks of twice daily topical application of 2% mupirocin ointment, his previous lesion of erythrasma (circled in white) had resolved (C). Wood lamp examination was negative for erythrasma on his left inguinal fold; there was no residual coral-pink fluorescence (circled in white) after six weeks of topical 2% mupirocin ointment therapy (D).

While he was lying on the table with legs apart for examination of the pedunculated lesion on his right thigh, his inguinal fold revealed an area of hypopigmentation surrounded by hyperpigmented scaling (Figure 1). Similar skin changes were also observed on the opposite inguinal fold (Figure 2). Wood lamp examination demonstrated bright coral-pink fluorescence on both inguinal folds (Figures 1-2).

Correlation of the clinical presentation and the Wood lamp examination finding established a diagnosis of erythrasma. The patient was instructed to apply 2% mupirocin ointment twice daily to the biopsy site and both areas of erythrasma for six weeks. The man returned after six weeks, and his biopsy site was healed. In addition, there were no residual skin lesions on his inguinal folds (Figures 1-2). He noted significant clearance after two weeks of therapy but continued his treatment regimen for the full six weeks. Wood lamp examination was also negative for coral-pink fluorescence, confirming clearance of the *Corynebacterium minutissimum* skin infection.

## Discussion

Erythrasma is a superficial infection of the skin typically caused by *Corynebacterium minutissimum*. It is more commonly found in diabetic and elderly populations. Indeed, erythrasma makes up 17.6% of bacterial skin infections in the elderly and 44% of toe web infections in individuals with diabetes [3, 4].

*Corynebacteria* are predominantly associated with pitted keratolysis of the feet, especially in those who are frequently barefoot. *Corynebacteria* can also cause trichobacteriosis of hair shafts in skin folds such as in the axillary region. Erythrasma is the least common of these three *Corynebacteria* skin infections [5].

The clinical presentation of erythrasma is characterized by asymptomatic lesions often in skin folds and moist areas. Red or brown hyperpigmented patches of skin with scaling and central hypopigmentation are usually observed. The lesions may also be slightly raised [6].

The most common diagnostic test for erythrasma is an examination with a Wood lamp. A Wood lamp is a black light source with a range of light emission between 320 and 400 nanometers. Peak emission occurs at 365 nanometers [7].

The diagnosis of erythrasma can be confirmed through observing coral-pink fluorescence during Wood lamp examination of the affected skin. Porphyrins, predominantly coproporphyrin III, made by *Corynebacteria* are the origin of this distinguishing fluorescence. Uroporphyrin I is also associated with the fluorescence of *Corynebacteria* under Wood lamp. Notably, false negatives can occur with Wood lamp examination if the site of the lesion was recently cleansed [1, 4].

Albeit rarely performed, a biopsy of the lesion can also establish a diagnosis of erythrasma. It is important for the clinician to alert the pathologist of the suspected condition because the specimen can mimic normal-appearing skin after hematoxylin and eosin staining. However, gram, Grocott’s methenamine silver, methylene blue, and periodic acid-Schiff (PAS) stains can help identify this bacteria. Biopsy of a lesion of erythrasma and special staining will reveal gram-positive (purple) coccobacilli in the superficial stratum corneum [5, 8].

The differential diagnoses of erythrasma, particularly in the groin region, include *Candida albicans* infection, dermatophyte infection, *Malassezia furfur* (pityriasis - also referred to as tinea - versicolor) infection, and terra firma-forme dermatosis (Table 1). These conditions can present as hyperpigmented and well-demarcated plaques; however, Wood lamp examination, potassium hydroxide (KOH) preparation findings, and treatment with 70% isopropyl alcohol can help distinguish between these lesions [1, 4, 6, 9-15].

**Table 1:** Differential diagnoses of erythrasma DDx - differential diagnosis; KOH - potassium hydroxide ^a^*Microspora *will also fluoresce green under Wood lamp. ^b^*Malassezia furfur* will also fluoresce yellow-gold under Wood lamp.

DDx	Candidiasis	Dermatophyte infection	Erythrasma	Pityriasis (tinea) versicolor	Terra firma-forme dermatosis (Duncan’s dirty dermatosis)
Etiology	Candida albicans (fungal)	Epidermophyton, Microsporum, or Trichophyton (fungal)	Corynebacterium minutissimum (bacterial)	Malassezia furfur (fungal)	Hyperkeratosis (no pathogen)
Clinical presentation	Dry, erosive, erythematous, and well-demarcated lesions.	Erythematous and scaly plaques.	Well-demarcated lesions with central hypopigmentation and peripheral hyperpigmented scaling.	Hyperpigmented and hypopigmented non-scaly lesions without sharp borders.	Dirt-like, hyperpigmented, and well-demarcated plaques.
Most common diagnostic test	KOH preparation with microscopic examination reveals pseudohyphae.	KOH preparation with microscopic examination reveals septate hyphae.^a^	Coral-pink fluorescence with Wood lamp examination.	KOH preparation with microscopic examination shows hyphae and spores.^b^	Removal of lesions with 70% isopropyl alcohol.
Histology	Pseudohyphae in the stratum corneum.	Septate hyphae in the stratum corneum.	Gram-positive coccobacilli in the stratum corneum.	Hyphae and clusters of spores in the stratum corneum (described as “spaghetti and meatballs”).	Hyperpigmented basal layer and compact orthokeratosis.
References	[6, 9]	[9-10]	[1, 4]	[6, 9-12]	[13-15]

Candidiasis is a fungal infection that can be identified using KOH preparation. If pseudohyphae are observed with KOH preparation, infection with *C. albicans* is most likely. In addition, Wood lamp examination will be negative for fluorescence [6, 9].

Dermatophyte infection is a fungal infection with either *Epidermophyton*, *Microsporum*, or *Trichophyton*. KOH preparation can confirm the diagnosis of a dermatophyte infection if septate hyphae are present in the stratum corneum. Some of these fungi can also fluoresce under Wood lamp [9-10].

Dermatophyte infections of hair, such as tinea capitis, may have a positive Wood lamp examination for ectothrix infection. In ectothrix infections, fungal organisms are located outside the hair shaft and allow for detection with Wood lamp examination. For example, *Microsporum* can fluoresce green [9-10].

Bacterial infection with *Malassezia furfur* is called pityriasis versicolor or tinea versicolor. Pityriasis versicolor can demonstrate yellow-gold fluorescence with Wood lamp. Histological examination may also help distinguish this pathogen and will reveal hyphae and small spores in the stratum corneum described as “spaghetti and meatballs”. However, the diagnosis is usually confirmed by examination of a KOH preparation of the lesion [6, 9-12].

Terra firma-forme dermatosis (Duncan’s dirty dermatosis) is unique among the differential diagnoses of erythrasma because there is no pathogen associated with this condition. Therefore, negative Wood lamp and KOH examinations can suggest terra firma-forme dermatosis. Histologic findings include hyperpigmentation in the basal layer and compact orthokeratosis [13-15].

Terra firma-forme dermatosis can be diagnosed by rubbing 70% isopropyl alcohol on the areas of hyperpigmentation. If the hyperpigmentation is removed, terra firma-forme dermatosis is the likely diagnosis. Identical to the diagnostic procedure, the treatment also involves the removal of all lesions with 70% isopropyl alcohol [13-15].

Several treatment options are available for erythrasma. Systemic oral treatment options include clarithromycin, erythromycin, and tetracycline (Table 2) [8, 16, 17]. Topical treatment options for erythrasma include clindamycin, fusidic acid, mupirocin, and Whitfield’s ointment (Table 3) [1, 16-20].

**Table 2:** Systemic treatments for erythrasma g - gram; mg - milligrams; GI - gastrointestinal; MOA - mechanism of action; QID - four times per day; Tx - treatment ^a^Chloramphenicol is an antibiotic that was previously used to treat erythrasma. It inhibits peptidyl transferase, disrupting bacterial protein synthesis. An oral 250 mg dosage four times daily can lead to the resolution of erythrasma in 14 days. However, the side effects, which include aplastic anemia, bone marrow suppression, and encephalopathy, deter from its use currently [17].

Tx	Clarithromycin	Erythromycin	Tetracycline
Dose	1 g single dose.	250 mg QID for 14 days.	250 mg QID for 14 days.
MOA	Macrolide antibiotic that inhibits protein synthesis by binding the 50S ribosomal subunit.	Macrolide antibiotic that inhibits protein synthesis by binding the 50S ribosomal subunit.	Antibiotic that inhibits protein synthesis by binding the 30S ribosomal subunit.
Benefits	Effective, fewer GI adverse events compared to erythromycin, and single-dose treatment (encourages compliance).	Effective and safer than chloramphenicol.^a^	Effective and safer than chloramphenicol.^a^
Possible adverse effects	Abdominal cramps, allergic reaction, dyspepsia, hearing loss, metallic taste in mouth, neutropenia, and ventricular arrhythmias.	Abdominal pain, allergic reaction, cholestatic hepatitis, dyspepsia, hearing loss, Steven-Johnson syndrome, and ventricular arrhythmias.	Allergic reaction, esophagitis, hemolytic anemia, phototoxic reaction, renal toxicity, and tooth discoloration.
References	[8, 16, 17]	[8, 16, 17]	[8, 17]

**Table 3:** Topical treatments for erythrasma EF-G: elongation factor G; g - grams; mg - milligrams; MOA - mechanism of action; tRNA - transfer ribonucleic acid; Tx - treatment ^a^Historically, studies found some topical antifungal agents to be successful in treating erythrasma. The twice daily application of 1% isoconazole nitrate and 0.1% diflucortolone valerate for five days was used as therapy with no reported side effects. Isoconazole (antifungal) inhibits ergosterol synthesis of the fungal cell membrane, and diflucortolone valerate (corticosteroid) dampens the inflammatory response. However, antifungals are currently not used to treat erythrasma [18]. ^b^Whitfield’s ointment consists of 12% benzoic acid and 6% salicylic acid. ^c^These side effects are uncommon with topical treatment yet have been noted with systemic therapy.

Tx^a^	2% clindamycin	2% fusidic acid	2% mupirocin	Whitfield’s ointment^b^
Dosage	Three times daily application for seven days.	Twice daily application for 14 days.	Twice daily application for 14 to 28 days.	Twice daily application for seven days.
MOA	Antibiotic that disrupts protein synthesis by binding the 50S ribosomal subunit.	Antibiotic that interferes with protein synthesis by binding EF-G.	Antibiotic that inhibits protein synthesis by binding isoleucyl-tRNA synthetase.	Benzoic acid: inhibits bacterial growth. Salicylic acid: keratolytic agent.
Possible adverse effects	Colitis, neutropenia, polyarthritis.^c^	Allergic reaction, ulceration.	None reported.	Allergic reaction, ulceration.
References	[17]	[16, 17, 19]	[1, 20]	[17]

Clarithromycin and erythromycin are macrolide antibiotics. Macrolides inhibit bacterial protein synthesis through reversibly binding the 50S bacterial ribosomal subunit. Oral treatment with 250 mg erythromycin four times daily can lead to the clearance of erythrasma in 14 days. A single one-gram dosage of clarithromycin is also effective; however, macrolides may be associated with gastrointestinal adverse events such as an upset stomach. Clarithromycin has greater bioavailability and a longer half-life compared to erythromycin. The milder gastric effects and single-dose treatment of clarithromycin can encourage better compliance and greater patient satisfaction [4, 8, 16, 17].

Tetracycline is a bacteriostatic agent that reversibly binds the 30S bacterial ribosomal subunit. It is usually well-tolerated but is associated with photosensitivity during therapy. In addition, it cannot be used in pregnant women and children because of tooth discoloration [8, 17].

Topical therapies for erythrasma are usually preferred over oral therapy because there are fewer and less severe adverse events associated with topical treatment. In fact, one study found topical fusidic acid cream to be more effective than oral clarithromycin and erythromycin in treating erythrasma. In addition, greater patient compliance has been observed with topical therapy compared to systemic therapy [8, 16].

Clindamycin is a bacteriostatic antibiotic that reversibly binds the 50S ribosomal subunit, disrupting protein synthesis. The topical application of this agent three times per day for one week can lead to complete eradication of erythrasma with no recurrence. Although systemic clindamycin therapy can be associated with colitis and neutropenia, these adverse events are rarely observed with topical treatment [17].

Fusidic acid is a bacteriostatic antibiotic that interferes with protein synthesis by binding elongation factor G involved in peptide elongation. Application of 2% fusidic acid cream twice daily for two weeks led to complete resolution of erythrasma for 96.8% of patients. Allergic reactions and ulceration were noted in some individuals with this therapy [16, 17, 19].

More recently, treatment with topical 2% mupirocin ointment has been shown to be effective in treating erythrasma. Mupirocin is an antibiotic that inhibits protein synthesis by binding isoleucyl-tRNA synthetase. Mupirocin ointment, which can be used to promote healing of a cryotherapy site in patients who have received treatment of actinic keratoses, can also be used to treat the infection with *Corynebacterium *[1, 20].

Nine men from the age of 32 years to 80 years were treated with topical 2% mupirocin ointment for two to four weeks. All patients observed the resolution of their erythrasma and had no recurrence at follow-up visits. In addition, there were no significant side effects associated with this treatment modality. The authors suggested that 2% mupirocin ointment be considered as first-line therapy for erythrasma [1].

Whitfield’s ointment contains 12% benzoic acid and 6% salicylic acid. These agents work together to inhibit bacterial growth and encourage sloughing off of skin cells (keratolysis). One study found twice daily application of Whitfield’s ointment for seven days as effective in treating erythrasma as 250 mg oral erythromycin or tetracycline taken four times per day for seven days [17].

We postulate that over-the-counter agents such as bacitracin, neomycin, and polymyxin may also be used to treat erythrasma. This hypothesis remains unexplored in studies. However, it could be a cost-effective and easily accessible topical treatment option for erythrasma.

## Conclusions

Erythrasma is a bacterial infection caused by *Corynebacterium minutissimum*. The clinical presentation and bright coral-pink fluorescence observed with Wood lamp examination can help to establish a diagnosis of erythrasma. Systemic treatments as well as topical treatments can be effective in treating this superficial skin infection. Topical therapy is routinely pursued due to fewer side effects and greater compliance compared to systemic therapies. Our patient was treated with topical 2% mupirocin ointment for six weeks and had noted nearly complete resolution within two weeks.
